# Molecular characterization of Anopheline (Diptera: Culicidae) mosquitoes from eight geographical locations of Sri Lanka

**DOI:** 10.1186/s12936-017-1876-y

**Published:** 2017-06-02

**Authors:** Thilini C. Weeraratne, Sinnathambi N. Surendran, Lisa J. Reimer, Charles S. Wondji, M. Devika B. Perera, Catherine Walton, S. H. P. Parakrama Karunaratne

**Affiliations:** 1 Department of Zoology, Faculty of Science, University of Peradeniya, Peradeniya, Sri Lanka; 2 Department of Zoology, Faculty of Science, University of Jaffna, Jaffna, Sri Lanka; 3 Liverpool School of Tropical Medicine, Liverpool, UK; 4 Regional Malaria Office, Kurunegala, Sri Lanka; 5 School of Earth and Environment, Faculty of Science and Engineering, University of Manchester, Manchester, UK; 6 National Institute of Fundamental Studies, Hantana, Kandy, Sri Lanka

**Keywords:** *Anopheles*, DNA barcoding, *COI*, ITS2, Mosquitoes, Taxonomy, Sri Lanka

## Abstract

**Background:**

Genus *Anopheles* is a major mosquito group of interest in Sri Lanka as it includes vectors of malaria and its members exist as species complexes. Taxonomy of the group is mainly based on morphological features, which are not conclusive and can be easily erased while handling the specimens. A combined effort, using morphology and DNA barcoding (using the markers *cytochrome c oxidase subunit I* (*COI*) gene and internal transcribed spacer 2 (ITS2) region, was made during the present study to recognize anophelines collected from eight districts of Sri Lanka for the first time.

**Methods:**

*Cytochrome c oxidase subunit I* and ITS2 regions of morphologically identified anopheline mosquitoes from Sri Lanka were sequenced. These sequences together with GenBank sequences were used in phylogenetic tree construction and molecular characterization of mosquitoes.

**Results:**

According to morphological identification, the field-collected adult mosquitoes belonged to 15 species, i.e., *Anopheles aconitus, Anopheles annularis, Anopheles barbirostris*, *Anopheles culicifacies, Anopheles jamesii, Anopheles karwari, Anopheles maculatus, Anopheles nigerrimus, Anopheles pallidus*, *Anopheles peditaeniatus*, *Anopheles pseudojamesi, Anopheles subpictus*, *Anopheles tessellatus*, *Anopheles vagus*, and *Anopheles varuna*. However, analysis of 123 *COI* sequences (445 bp) (16 clades supported by strong bootstrap value in the neighbour joining tree and inter-specific distances of >3%) showed that there are 16 distinct species. Identity of the morphologically identified species, except *An. subpictus,* was comparable with the DNA barcoding results. *COI* sequence analysis showed that morphologically identified *An. subpictus* is composed of two genetic entities: *An. subpictus* species A and species B (inter-specific K2P distance 0.128). All the four haplotypes of *An. culicifacies* discovered during the present study belonged to a single species. ITS2 sequences (542 bp) were obtained for all the species except for *An. barbirostris, An. subpictus* species B, *An. tessellatus*, and *An. varuna*. Each of these sequences was represented by a single species-specific haplotype.

**Conclusions:**

The present study reflects the importance and feasibility of *COI* and ITS2 genetic markers in identifying anophelines and their sibling species, and the significance of integrated systematic approach in mosquito taxonomy. Wide distribution of malaria vectors in the country perhaps indicates the potential for re-emergence of malaria in the country.

**Electronic supplementary material:**

The online version of this article (doi:10.1186/s12936-017-1876-y) contains supplementary material, which is available to authorized users.

## Background

Species identification is essential for many biological studies. Morphological characters and DNA barcoding are two major approaches used in species identification. The modern system of taxonomy, referred to as ‘DNA barcoding’, which is based on molecular techniques, has become increasingly popular as it potentially produces results with very high precision and accuracy within a short period of time, compared to traditional morphology-based taxonomy. DNA barcoding is currently used as a guide in biodiversity conservation and management programmes due to its ability to confirm the identity of organisms, to infer the presence of new/endangered/invasive/cryptic species and to estimate genetic diversity among individuals of a species [1].

Due to their importance in disease transmission, mosquitoes are one of the most intensely barcoded group of insects, although many species are still to be identified and barcoded [2]. Further, mosquito species may exist as species complexes and sibling species of a given complex often show different feeding behaviour, biting habits, vector competence, distribution, and thereby different vectorial capacities. Accurate and precise identification of mosquito species and sibling species is vital in planning vector control strategies. Since these sibling species cannot be distinguished by external morphological features, molecular taxonomy is required to obtain reliable information about the vector.

The most commonly used molecular markers/barcode regions for mosquito barcoding studies are the *cytochrome c oxidase subunit I* (*COI*) gene located in the mitochondrial genome (mtDNA) followed by the internal transcribed spacer 2 (ITS2), a region from the nuclear ribosomal DNA. Several workers have used *COI* as the only marker in mosquito species recognition and in investigating their molecular evolution [3–9]. However, some studies have shown that mosquito identification through *COI* alone is not always sufficient to make precise conclusions and the need for genetic examination using analysis of faster evolving regions, such as ITS2, has been emphasized [8]. ITS2 regions alone have been used in distinguishing closely related mosquito species belonging to various genera such as *Anopheles* [10], *Culex* [11] and *Aedes* [12]. Moreover, it has been indicated that more reliable information about species could be obtained if several molecular markers are used simultaneously [13, 14].

The mosquito fauna in Sri Lanka comprises 141 species belonging to 17 genera [15–17]. Out of these, 16.31% (23 species) are anophelines. An accurate and detailed species characterization of members of this genus is required. In Sri Lanka, major malaria vector species *Anopheles culicifacies* and *Anopheles subpictus* exist as species complexes [18–20]. Among the two *An. culicifacies* species B and E found in Sri Lanka, species E is the primary vector of malaria in the country [18]. *Anopheles subpictus* (four sibling species viz. A, B, C and D) is considered the major vector of malaria in Jaffna district and the secondary vector in other parts of the country [21]. Several studies have found higher malaria sporozite rates in *An. subpictus* than *An. culicifacies* during the peak transmission period in Jaffna district [22, 23]. Further, *Anopheles aconitus*, *Anopheles annularis*, *Anopheles barbirostris*, *Anopheles nigerrimus*, *Anopheles pallidus*, *Anopheles peditaeniatus*, *Anopheles tessellatus*, *Anopheles vagus*, and *Anopheles varuna* are considered potential vectors of malaria [24, 25].

In Sri Lanka, mosquito identification has mainly relied on taxonomic keys based on external morphological features of adult and larval stages. However, a few studies have attempted to use DNA barcodes to describe a few species, i.e., *An. culicifacies* [26, 27], *An. subpictus* [18], *An. barbirostris* [28], and *Anopheles sundaicus* [29], from limited geographic locations. The country urgently needs a well planned molecular and morphology-based taxonomy to characterize its anopheline population. The present study aims to provide both morphological and molecular taxonomic (DNA barcoding) information about *Anopheles* mosquitoes collected from different geographical areas of Sri Lanka. The importance of species identification using two molecular markers *COI* and ITS2 is also discussed.

## Methods

### Study sites

**Fig. 1:**
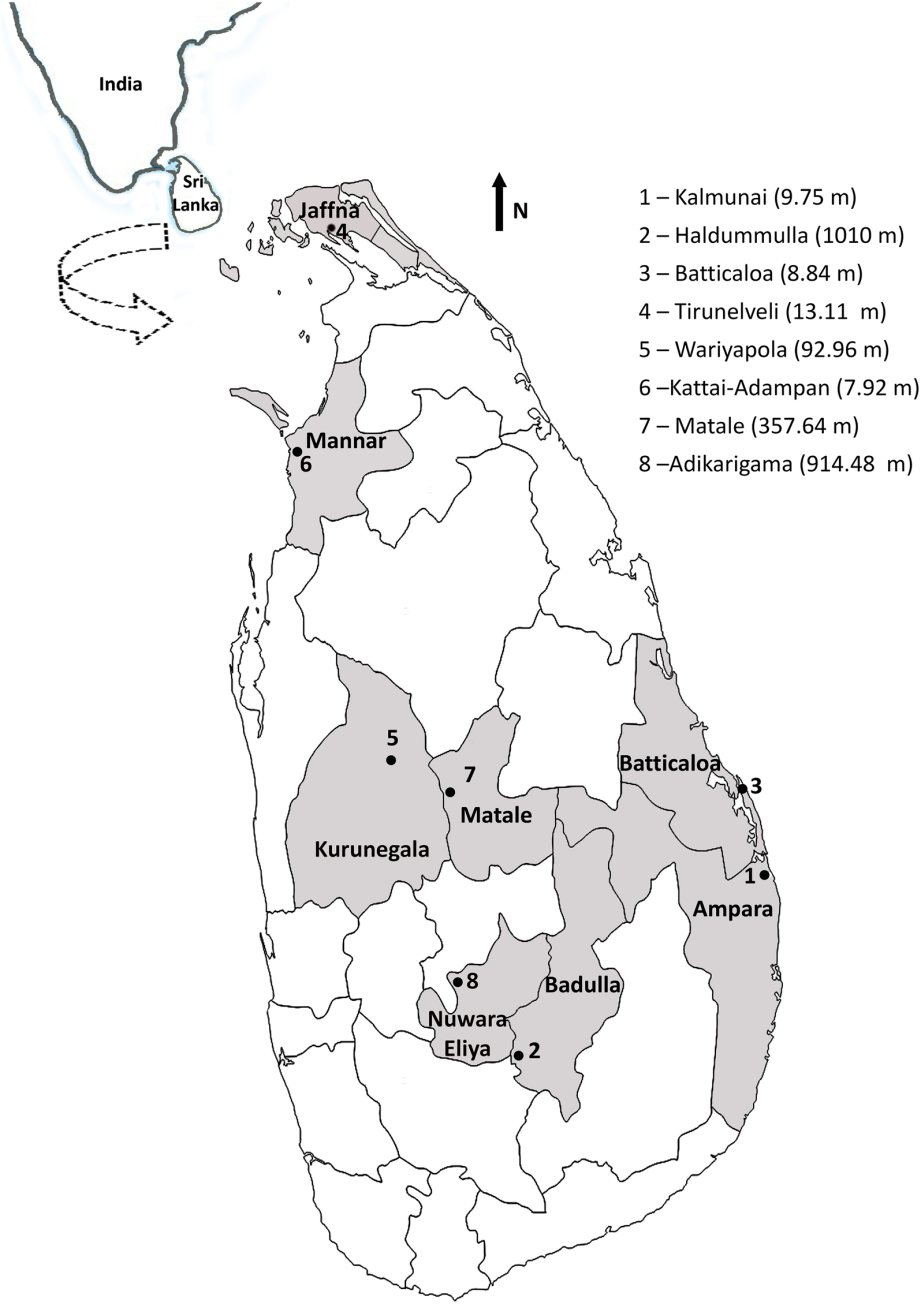
Map of Sri Lanka showing the districts and the collection sites (*1*–*8*) from which mosquitoes were collected for the DNA barcoding study (elevations are given in *parentheses*)

Mosquitoes were collected from eight study sites located in eight districts in Sri Lanka: Kalmunai in Ampara district, Haldummulla in Badulla district, Batticaloa in Batticaloa district, Tirunelveli in Jaffna district, Wariyapola in Kurunegala district, Matale in Matale district, Kattai-Adampan in Mannar district, and Adikarigama in Nuwara-Eliya district (Fig. 1). These sites are located in five different climatic zones, i.e., Adikarigama in up-country wet zone (>900 m elevation, >2500 mm rainfall); Matale in mid-country wet zone (300–900 m elevation, >2500 mm rainfall); Haldummulla in up-country intermediate zone (>900 m elevation, 1750–2500 mm rainfall); Wariyapola in low-country intermediate zone (0–300 m elevation, 1750–2500 mm rainfall); Kalmunai, Batticaloa, Tirunelveli, Katti-Adampan in low-country dry zone (0–300 m elevation, <1750 mm rainfall, with a distinct dry period) (Fig. 1). Habitat types of each study site are presented in Additional file 1.

### Mosquito collection

Adult mosquitoes were collected using cattle-baited traps and backpack aspirators. Dried specimens were used in morphological and molecular identifications. Morphological identifications to species or complex level were done using standard taxonomic keys [16, 30]. Voucher samples of specimens from each species were mounted using standard techniques for a reference collection at the Department of Zoology, Faculty of Science, University of Peradeniya, Sri Lanka. The reminder were dried and stored for molecular characterization.

### DNA extraction, polymerase chain reaction (PCR) and sequencing

Genomic DNA was extracted from head and thoracic regions of each individual using nexttec™ DNA Isolation Kits (Nexttec Biotechnologies GmbH, Leverkusen, Germany), according to the manufacturer’s protocol. DNA was extracted from a maximum of ten individuals from each morphologically identified species, depending on the availability.

A region of the *COI* gene was amplified using forward primer C1-J-1718 (5′-GGAG GATTTGGAAATTGATTAGTTCC-3′) and reverse primer C1-N-2191 (5′-CCCGGTAAAATTAAAATATAAACTTC-3′) [31] and the ITS2 was amplified using forward primer 5′-ATCACTCGGCTCATGGATCG-3′ and reverse primer 5′-ATGCTTAAATTTAGGGGGTAGTC-3′ [32]. Each amplification was performed in 15 µl that included 1 µl DNA template, 1.5 µl 10× KAPA buffer A, 0.12 µl KAPA taq, 0.12 µl 2.5 mMd NTPmix, 0.75 µl 50 mM Mgcl_2_, 0.51 µl each primer (10 mmol), and 10.49 µl ddH_2_O. The PCR parameters were 95 °C for 5 min and 35 cycles of 94 °C for 30 s, 51 °C (for *COI*)/55 °C (for ITS2) for 40 s and 72 °C for 45 s, followed by a final extension step of 72 °C for 10 min. PCR products were run in 1.5% agarose gel stained with Medori green and visualized in a gel imaging system.

PCR products showing positive clear bands were purified using QIAquick^®^ PCR Purification kits according to the manufacturers’ protocol. A maximum of three PCR positive samples of each species from each district were sequenced bidirectionally at Source Bioscience, Nottingham, UK.

### DNA sequence analysis

The trace files/chromatograms of *COI* and ITS2 sequences were manually edited using BioEdit software. Sequences of low quality were excluded during data analysis. Consensus sequences were aligned using Clustal W in BioEdit software [33]. Once the alignment was completed, species identification was confirmed by comparison to publicly available sequence data in GenBank using BLAST [34] and the barcode of life database (BOLD) interface [35]. The number of parsimony informative sites, number of variable sites, number of haplotypes, haplotype diversity, and GC content were analysed using the DNA Sequences Polymorphism software (dnaSP) (Version 5.1.10). Mega version 6.0 was used to calculate intra-specific and inter-specific pairwise sequence divergence using the Kimura-2 parameter distance model [36]. Neighbour joining (NJ) phylogenetic trees of *COI* and ITS2 sequences were constructed in MEGA 6.0 using Kumura-2 parameter distances. Branch support of NJ trees was assessed by bootstrapping with 1000 replicates. Codon positions included 1st + 2nd + 3rd + non-coding regions. All the haplotype sequences of *COI* and generated ITS2 sequences were deposited in GenBank and the accession numbers are given in Additional file 2.

## Results

According to morphological identification, the wild mosquito samples collected from all eight study sites belonged to 15 anopheline species: *Anopheles aconitus*, *An. annularis*, *An. barbirostris*, *An. culicifacies*, *An. jamesii*, *An. karwari*, *An. maculatus*, *An. nigerrimus*, *An. pallidus*, *An. peditaeniatus*, *An. pseudojamesi*, *An. subpictus*, *An. tessellatus*, *An. vagus,* and *An. varuna*. The distributions of species among the study sites are given in Table 1. *Anopheles peditaeniatus* and *An. vagus* were the most widespread, occurring at seven out of eight study sites. *Anopheles peditaeniatus* was absent from Adikarigama in Nuwara-Eliya district site while *An. vagus* was absent from Kattai-Adampan in Mannar district. *Anopheles aconitus* and *Anopheles pseudojamesi* were present only in Haldummulla (Badulla district) while *An. maculatus* and *An. tessellatus* were recorded only from Adikarigama (Nuwara-Eliya district) and Wariyapola (Kurunegala district), respectively (Table 1). Anopheline adults were collected using two different collection methods: cattle-baited traps and backpack aspirators. Cattle-baited traps were positive for all the above species whereas backpack aspirators were positive only for *An. culicifacies*, *An. jamesii, An. varuna*, and *An. subpictus*.

**Table 1:** Statistical parameters of the *COI* sequences and the distribution of species in the study sites

Species	n	h	Study sites	Mean pairwise distance	Pairwise distance range	Haplotype diversity
*An. aconitus*	2	2	Haldummulla	0.003 ± 0.003	–	1.000 ± 0.500
*An. annularis*	3	3	Haldummulla, Tirunelveli	0.010 ± 0.004	0.012 to 0.009	1.000 ± 0.272
*An. barbirostris*	5	1	Haldummulla, Wariyapola, KattaiAdampan	0.000 ± 0.000	–	0.000 ± 0.000
*An. culicifacies*	5	4	Haldummulla, Tirunelveli, Adikarigama	0.012 ± 0.004	0.000 to 0.018	0.900 ± 0161
*An. jamesii*	13	7	Haldummulla, Tirunelveli, Wariyapola, KattaiAdampan, Matale	0.001 ± 0.001	0.000 to 0.006	0.731 ± 0.133
*An. karwari*	2	1	Wariyapola, Batticoloa	–	–	0.000 ± 0.000
*An. maculatus*	3	3	Adikarigama	0.012 ± 0.005	0.006 to 0.017	1.000 ± 0.272
*An. nigerrimus*	6	5	Tirunelveli, KattaiAdampan, Matale	0.009 ± 0.003	0.003 to 0.018	0.933 ± 0.122
*An. pallidus*	16	4	Kalmunai, Haldummulla, Batticoloa, Tirunelveli, Wariyapola	0.002 ± 0.001	0.000 to 0.006	0.525 ± 0.137
*An. peditaeniatus*	19	10	Kalmunai, Haldummulla, Batticoloa, Tirunelveli, Wariyapola, KattaiAdampan, Matale	0.007 ± 0.002	0.000 to 0.023	0.883 ± 0.056
*An. pseudojamesi*	2	1	Haldummulla	0.000 ± 0.000	–	0.000 ± 0.000
*An. subpictus* species A	24	16	Kalmunai, Haldummulla, Batticoloa, Wariyapola, KattaiAdampan	0.006 ± 0.002	0.000 to 0.018	0.938 ± 0.001
*An. subpictus* species B	2	2	Tirunelveli	0.002 ± 0.002	–	1.000 ± 0.250
*An. tessellatus*	3	3	Wariyapola	0.006 ± 0.003	–	1.000 ± 0.272
*An.vagus*	13	12	Kalmunaim, Haldummulla, Batticoloa, Tirunelvei, Wariyapola, Matale, Adikarigama	0.009 ± 0.003	0.000 to 0.017	0.987 ± 0.035
*An. varuna*	5	5	Tirunelveli, Wariyapola	0.014 ± 0.004	0.009 to 0.029	1.000 ± 0.126

Intra-specific distances were calculated using Kimura 2-parameter distance algorithm

*n* total number of *COI* sequences, *h* haplotype number

**Fig. 2:**
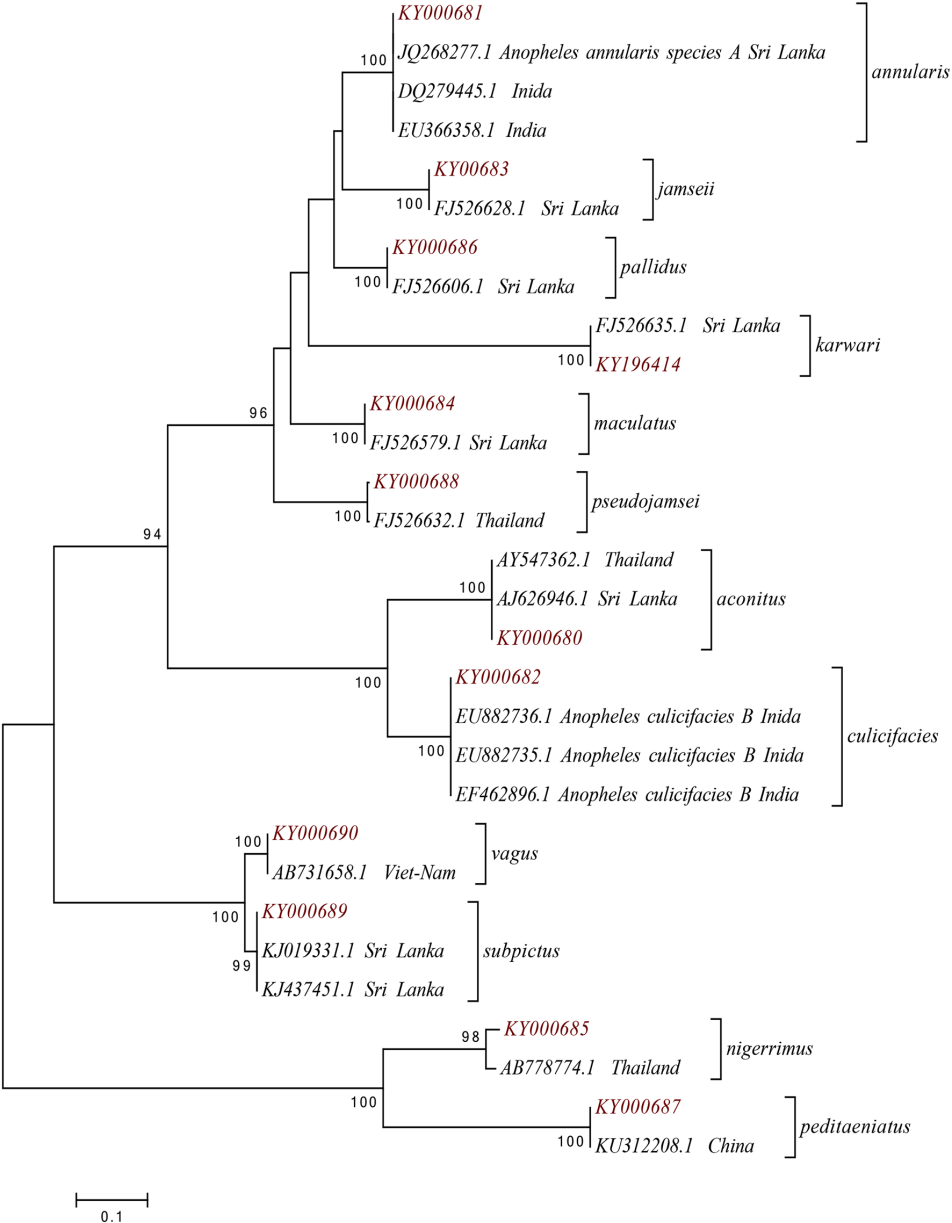
Neighbour joining phylogenetic tree (based on Kimura 2-parameter genetic distance model) using 12 ITS2 sequences generated during the present study (*red label*) and sequences retrieved from the GenBank (*black label*). Only node support greater than 90% is shown

A total of 123 *COI* sequences were obtained for all 15 anopheline species and 79 *COI* haplotypes were identified. *COI* sequences with a fragment size of 445 bp were used in the phylogenetic analysis. ITS2 sequences were obtained only for 12 species and each of those species had only a single haplotype (12 species-specific ITS2 haplotypes). The ITS2 sequences generated for *An. barbirostris*, *An. tessellatus* and *An. varuna* were not included in the analysis since they were not in good quality. The length of ITS2 fragment varied from 398 bp in *An. maculatus* to 506 bp in *An. nigerrimus*. Fragment sizes obtained from all the species are given in Additional file 2 together with the GenBank accession numbers where the sequences were deposited. The sequence match between these haplotypes and the closest publicly available sequence was always 99–100%. The NJ tree constructed using the 12 ITS2 sequences is shown is Fig. 2. The NJ tree formed 12 distinct clades, each representing single species (supported by 98–100% bootstrap values).

*Cytochrome c oxidase subunit I* sequences were AT rich and with an overall G+C content of 0.315. According to polymorphism analysis, there were 149 variable bases (33.9%) of which 130 were parsimony informative and only 17 were singleton mutations. Out of all the nucleotide variations, 78.5% was observed at the third codon position with 117 variable sites and eight singleton mutations. The first position had 25 variable sites and five singleton mutations accounting for 16.8% of all the variable sites. The rest of the variation (4.7%) was observed at the second codon position.

**Fig. 3:**
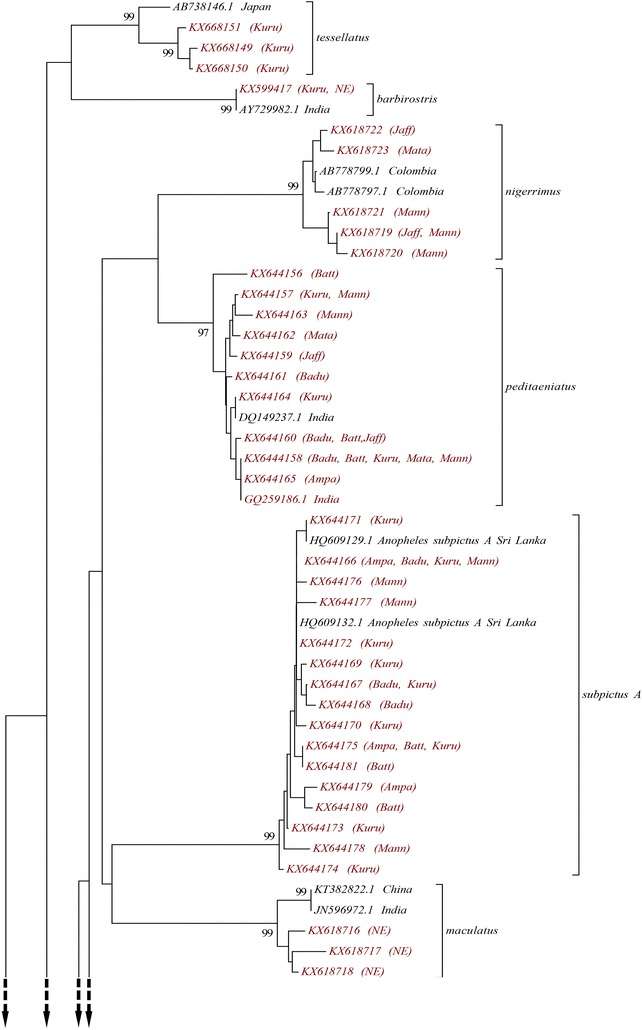
Neighbour joining phylogenetic tree (based on Kimura 2-parameter genetic distance model) of *COI* sequences of all 79 haplotypes of anopheline species collected from Sri Lanka during the study (*red label*) and the sequences retrieved from the GenBank (*black label*). *Armigeres subalbatus* and *Culex tritaeniorhynchus* were used as the out-groups. Only node support greater than 90% is shown. The study site/s from which each haplotype was collected are given in parentheses (*Ampa* Ampara, *Badu* Badulla, *Batt* Batticoloa, *Jaff* Jaffna, *Kuru* Kurunegala, *Mann* Mannar, *Mata* Matale, *NE* Nuwara-Eliya)

**Fig. 4:**
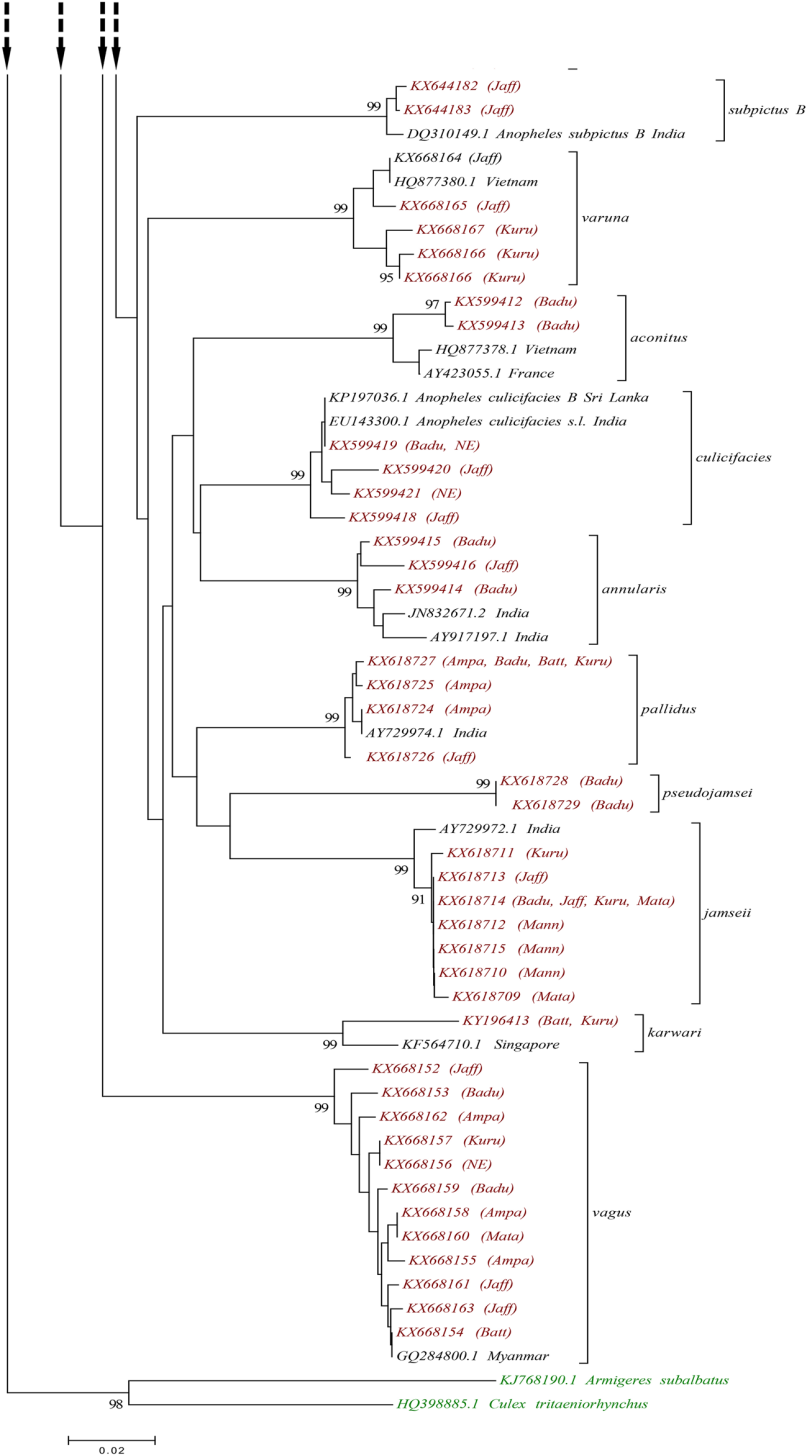
Neighbour joining phylogenetic tree (based on Kimura 2-parameter genetic distance model) of *COI* sequences of all 79 haplotypes of anopheline species collected from Sri Lanka during the study (*red label*) and the sequences retrieved from the GenBank (*black label*). *Armigeres subalbatus* and *Culex tritaeniorhynchus* were used as the out-groups. Only node support greater than 90% is shown. The study site/s from which each haplotype was collected are given in parentheses (*Ampa* Ampara, *Badu* Badulla, *Batt* Batticoloa, *Jaff* Jaffna, *Kuru* Kurunegala, *Mann* Mannar, *Mata* Matale, *NE* Nuwara-Eliya)

All the 15 morphologically identified *Anopheles* species were grouped into 16 distinct clades in the NJ tree constructed using *COI* sequences. All of these clades were strongly supported by 99% bootstrap value except *An. peditaeniatus*, which was supported by a 97% bootstrap value. For 15 species, the specimens of each species clustered into a single clade but the specimens of *An. subpictus* fell into two separate clades, as shown in Figs. 3 and 4.

**Fig. 5:**
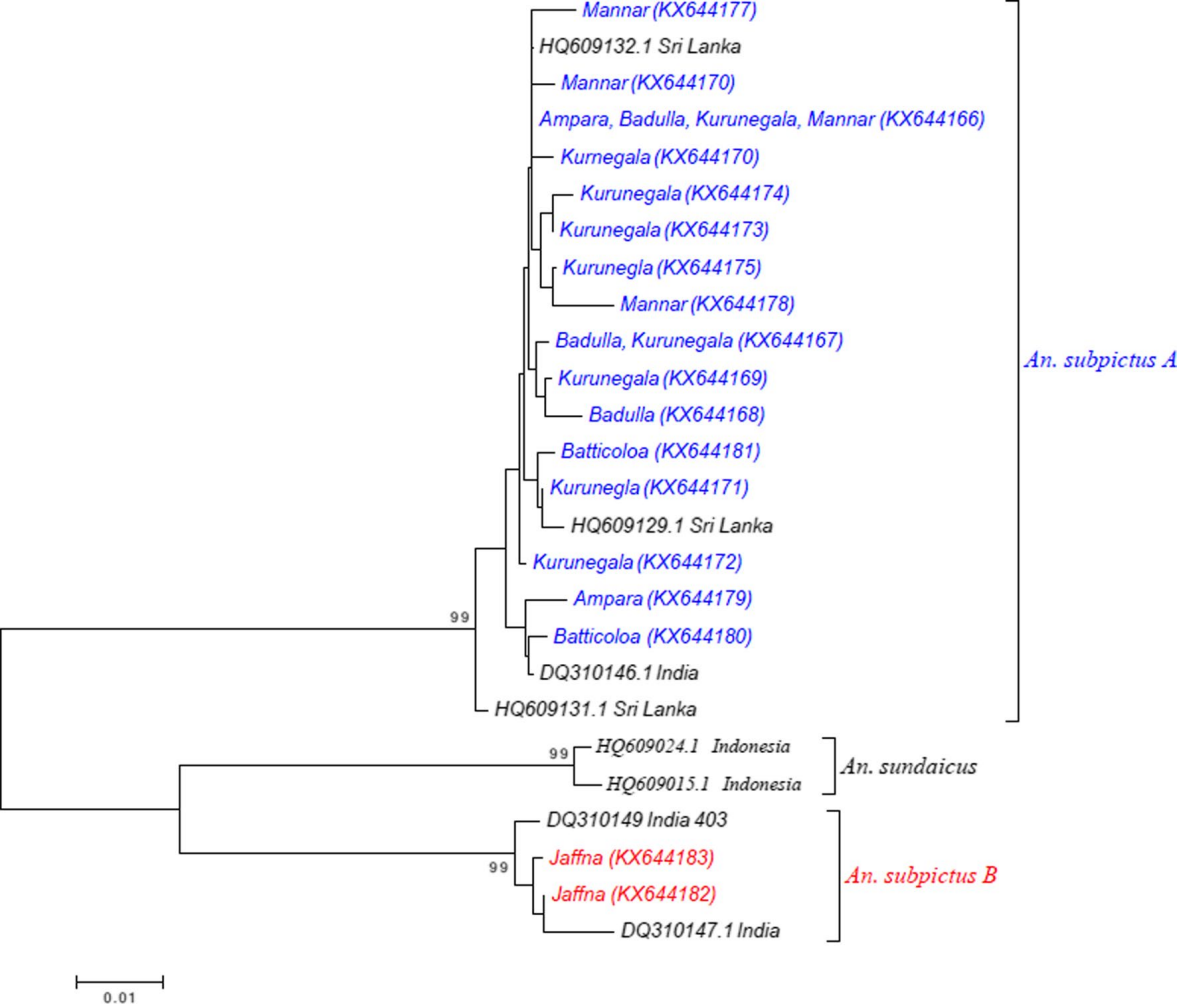
Neighbour joining phylogenetic tree (based on Kimura 2-parameter genetic distance model) constructed using *COI* sequences of *Anopheles subpictus* species A (16 haplotypes in *blue*) and *Anopheles subpictus* species B (2 haplotypes in *red*) obtained during the study and *COI* sequences of *Anopheles sundaicus* retrieved from the GenBank. Only the node support greater than 90% is shown

Figure 5 shows the phylogenetic tree constructed using all the 18 *An. subpictus COI* haplotypes obtained during the current study and the sequences retrieved from GenBank. Separation of *An. subpictus* haplotypes into two distinct clusters indicates the presence of two different genetic entities of *An. subpictus* in Sri Lanka. The two haplotypes of *An. subpictus* from Jaffna (KX644182 and KX644183) formed a separate clade together with *An. subpictus* species B from India (DQ310147.1 and DQ310149.1) sharing 99% sequence similarity according to BLAST search results. All the other 16 *An. subpictus* specimens from other areas clustered into one group (bootstrap value of 100%) with *An. subpictus* species A sequences from Sri Lanka (HQ609132.1, HQ609129.1) and India (DQ310146.1).

Out of these 16, six species, i.e., *An. aconitus*, *An. annularis*, *An. maculatus*, *An. subpictus* species B, *An. tessellatus*, and *An. varuna,* showed a haplotype diversity of 1.000. The lowest haplotype diversity was among *An. pallidus* specimens (0.525 ± 0.137). No polymorphism was shown by *An. barbirostris, An. karwari* and *An. pseudojamesi* (Table 1).

The overall mean Kimuara-2 parameter genetic distance between these 14 *Anopheles* species was 0.106 ± 0.011. The intra-specific K2P genetic distances was <2%, as given in Table 1. The maximum mean intra-specific distance was for *An. varuna* (1.4%) followed by *An. culicifacies* and *An. maculatus* (1.2%), while *An. jamesii* had the minimum intra-specific K2P distance of 0.1%.

The inter-specific K2P genetic distances between all the species were more than 2%. The highest K2P value was between *An. jamesii* and *An. barbirostris* (17.2%) and least was between *An. peditaeniatus* and *An. nigerrimus* 7.2%. The inter-specific distances between *An. subpictus* species A and species B was 12.8% (Table 2).

**Table 2:** Inter-specific (below the diagonal) and the mean intra-specific distances (along the diagonal) of *COI* sequences

	*aco*	*ann*	*bar*	*cul*	*jam*	*kar*	*mac*	*nig*	*pal*	*ped*	*psej*	*subA*	*subB*	*tes*	*vag*	*var*
*aco*	0.003															
*ann*	0.114	0.010														
*bar*	0.143	0.134	0.000													
*cul*	0.088	0.077	0.120	0.012												
*jam*	0.122	0.123	0.172	0.116	0.001											
*kar*	0.142	0.114	0.126	0.115	0.137	0.000										
*mac*	0.134	0.131	0.127	0.114	0.109	0.126	0.012									
*nig*	0.123	0.140	0.122	0.114	0.140	0.142	0.115	0.009								
*pal*	0.092	0.080	0.125	0.089	0.106	0.097	0.108	0.115	0.002							
*ped*	0.127	0.117	0.119	0.111	0.126	0.128	0.118	0.072	0.102	0.007						
*psej*	0.147	0.101	0.171	0.120	0.107	0.143	0.128	0.140	0.088	0.143	0.000					
*subA*	0.139	0.124	0.133	0.112	0.129	0.144	0.120	0.135	0.119	0.097	0.151	0.002				
*subB*	0.130	0.118	0.133	0.112	0.137	0.135	0.139	0.139	0.132	0.130	0.141	0.128	0.006			
*tes*	0.131	0.134	0.093	0.101	0.153	0.135	0.122	0.114	0.132	0.098	0.159	0.116	0.145	0.006		
*vag*	0.155	0.135	0.134	0.149	0.139	0.126	0.132	0.126	0.138	0.111	0.155	0.134	0.130	0.123	0.009	
*var*	0.113	0.111	0.135	0.090	0.116	0.131	0.148	0.141	0.107	0.111	0.143	0.125	0.117	0.138	0.125	0.014

Distances were calculated using Kimura 2-parameter distance algorithm

*aco An. aconitus, annu An. annularis, bar An. barbirostris, cul An. culicifacies, jam An. jamesii, kar An. karwari, mac An. maculatus, nig An. nigerrimus, pal An. pallidus, ped An. peditaeniatus, psej An. pseudojamesi, subA An. subpictus species A, subB An. subpictus species B, tes An. tessellatus, vag An. vagus, var An. varuna)*

Translation of nucleotide data to amino acids resulted in an alignment of 144 amino acids. The 95th amino acid has changed from isoleucine to valine (or I arrow V) in all specimens of *An. peditaeniatus*, *An. barbirostris*, *An. nigerrimus*, *An. tessellatus*, *An. vagus*, and *An. subpictus* species A. All the other species shared identical AA amino acid sequences.

## Discussion

The present study provides morphological and molecular characterization of anopheline species from eight districts across different climatic zones in Sri Lanka. Morphology-based taxonomy recognized 15 *Anopheles* species while DNA barcoding was able to recover two sibling species within the morphologically identified *An. subpictus* specimens, bringing the total species count to 16.

According to the morphological identification results, 65.22% of morphologically identified *Anopheles* species present in Sri Lanka (15 species out of 23) were found in these eight study sites. Although Sri Lanka is a small island (65,525 sq km), a sharp increase in elevation can be seen from sea level to the centre of the country (highest elevation 2524 m). Elevation has been identified as one of the major factors that influences the vegetation pattern of a given region as it affects climatic factors. Western, southern and central regions situated in the wet and intermediate zones experience heavy showers for a long period of the year with a short dry period. Northern and eastern areas (dry zone) receive relatively low rains and undergo a prolonged dry season. Climate affects the prevalence and abundance of mosquito populations in different geographical areas of the country.

Prevalence of Sri Lankan mosquito species, including anophelines, has shown a significant seasonal and site-related density variation and weather has been identified as the most responsible macro-ecological factor [37–39]. Further, mosquito abundance declines during the dry season compared to the monsoon season with high rainfall [39]. During the present study, *An. peditaeniatus* and *An. vagus,* two potential vectors of malaria were common to almost all the study sites. These two are paddy field breeders and paddy fields were common in all the study sites [39, 40]. Absence of *An. peditaeniatus* from Adikarigama site and *An. vagus* from Kattai-Adampan site may be due to seasonal variations at the time of mosquito collection.

Alternatively, *An. aconitus*, *An. maculatus*, *An. pseudojamesi*, and *An. tessellatus* were the least common mosquitoes, and each was restricted to a single locality. *An. maculatus* is considered as a stream breeder adapted to survive mainly at high altitudes near hilly or mountainous areas [41]. A previous mosquito survey conducted in Kandy district in the up-country wet zone of Sri Lanka reported *An. maculatus* as the dominant species [42]. *An. aconitus* is also considered a species that breeds at high altitudes [43]. In the present study, both these species were found at study sites with highest elevations: *An. maculatus* from Adikarigama (614.48 m) and *An. aconitus* from Haldummulla (1010 m). *An. pseudojamesi* and *An. tessellatus* are rare and least abundant anophelines in Sri Lanka [44]. It has been reported that *An. tessellatus* commonly breeds in locations similar to Wariyapola study site, which had ample sunlight with less vegetation [45]. The presence of the major and potential vectors of malaria throughout the country regardless of environmental conditions shows the potential threat of malaria re-emerging in the country where it has at present been eliminated.

As mosquitoes are vectors of many human diseases, accurate identification is essential in implementing vector control programmes [6, 46]. During the current study mitochondrial gene *COI* and nuclear ribosomal region ITS2 were used to confirm the identity of morphologically identified species. Presence of only a single ITS2 haplotype for each species reflects the importance of use of ITS2 in barcoding studies as the ITS2 sequences are species specific [47, 48].

As was previously mentioned, all the species were confirmed by comparing the sequences with the sequences already available in the GenBank and BOLD system and all the sequences had a maximum identity value between 99 and 100%. NJ trees using K2P substitution model are the most widely constructed phylogenetic trees used in many barcoding studies, especially on mosquitoes, to obtain more accurate recognition of the targeted group [3, 6, 9, 49]. Accordingly, the same methodology was applied during this study to increase the reliability of the results. Based on *COI* sequence similarity, individuals that were morphologically grouped into species, except *An. subpictus,* were grouped into 14 distinct clades, with strong bootstrap support in the NJ phylogenetic tree confirming the results of morphological identification.

Based on genetic distance estimates for *COI* sequences, 2–3% sequence divergence or pairwise distance between species (inter-specific divergence) has been considered the threshold to differentiate two species [50, 51]. This has been used as the cutoff in many mosquito studies to distinguish closely related mosquito species [3, 6, 9]. The inter-specific K2P distances of the *COI* sequences of all the species exceeds 3% (0.03), verifying that they precisely belong to different species. This indicates the significance of the *COI* region as a marker in identifying mosquitoes at species level.

According to phylogenetic tree analysis, all the morphologically identified *An. subpictus* samples comprise two genetic entities as they separate into two different clades supported by 99% bootstrap value (a clade containing samples from Jaffna and a clade including samples from all the other localities). Further, the K2P distances calculated between these two genetically different *An. subpictus* samples were >3%. This verifies that the *An. subpictus* samples, which were morphologically identified as a single species, belong to two different species. Further, the present analysis revealed that all the samples from Jaffna are *An. subpictus* species B while all the other samples are *An. subpictus* species A. *An. subpictus* species B has been reported as the most dominant *Anopheles* species in both coastal (brackish water) and inland (fresh and nearly saline water) of Jaffna district [23] and it is more prevalent in coastal areas [18, 52, 53]. According to the results of this study, *An. subpictus* species B was found only from Jaffna district. This might be due to the high abundance of this species in the district and presence of water bodies with brackish and saline water. *An. subpictus* from other areas were *An. subpictus* species A. This species is known to breed mainly in inland freshwater bodies [52, 53]. Hence, it was found from study sites within the country and also from Batticaloa and Kalmunai with coastal areas. Higher prevalence of *An. subpictus* species A than species B in these areas might be the reason behind this observation. The DNA barcoding study by Kumar et al. [9], reported a 11.3% difference between these two sibling species in India and it was slightly higher (12.8%) in the present study. A molecular (using *COI* and ITS2) and morphology (identified based on the number of egg ridges)-based study showed that, although there are four sibling species *of An. subpictus* (A, B, C, D) according to egg morphology, the Sri Lankan subpictus complex actually comprise two genetically distinct species: *An. subpictus* species A and species B [18]. The phylogenetic tree analysis of this study showed that *An. subpictus* species B is more closely related to *An. sundaicus* than *An. subpictus* species A, as observed in previous studies [18]. This indicates the considerable genetic distance between these two species and supports the above statement. More importantly the current study reveals the importance of DNA barcoding in identifying sibling species/closely related species that may have different vectorial capacities.

*Anopheles culicifacies* species E is the primary vector of malaria in Sri Lanka and *An. culicifacies* species B is a non- or poor vector [18]. All the four haplotypes of *An. culicifacies* discovered during the present study belong to a single species. Although several studies have attempted to use DNA barcodes *COI*, *COII*, ITS2 and D3 in characterizing *An. culicifacies* species complex in Sri Lanka, none could accurately identify sibling species of this vector [27]. This may be due to the unavailability of all the sibling species in their collections or due to the inefficiency of the molecular-based approaches in recognizing sibling species of *An. culicifacies* species complex.

A study limited only to Jaffna district revealed the presence of *An. annularis* species A in Sri Lanka [53]. The present study shows no inter-specific variation among *An. annularis* collected from two different climatic zones: dry zone (Jaffna) and wet zone (Badulla). This confirms the presence of *An. annularis* species A in Sri Lanka. A phylogenetic analysis of medically important mosquitoes in India has observed the presence of the same substitutions and indels in the ITS2 sequences of Indian *An. annularis* species A and *An. annularis* specimens from Sri Lanka, further confirming the sibling status of *An. annularis* in Sri Lanka [4]. Moreover, *An. annularis* species A is a malaria vector species in India, making it a very important vector species that needs attention [53].

The intra-specific divergence obtained during the study varied between 0 and 1.4%, showing that the *COI* divergence between the haplotypes of a single species is lower than the threshold value. DNA barcoding studies conducted in Pakistan and China reported 0–2.4 and 0–1.67% divergence ranges, respectively, similar to observations of the current study [5, 7, 54]. An intra-specific divergence of 1.84% has reported among the haplotypes of *An. pallidus* in India [9]. The highest intra-specific divergence was observed for *An. varuna* (1.4%) which was based on a comparison of a single sequence obtained from each of two distant localities: Wariyapola in Kurunegala and Tirunelveli in Jaffna district. The two study sites are located in two climatic zones and the Wariyapola site is at a higher elevation (low-country intermediate zone) than the Tirunelveli site (dry zone) (≈80 m difference). A study in India has observed very high intra-specific divergence of 4.9% among *Anopheles stephensi* samples and variation in the climate of the areas was found as the main reason for this high intra-specific divergence [4]. Further, Murugan et al. [4] suggest increasing the sample size and expanding the geographical area of collection to clarify this answer. *Anopheles varuna* was collected only from two study sites located at different elevations and climatic zones and sequences obtained from a relatively small sample would have been the reason behind this high intra-specific divergence, which needs further investigation. The K2P inter-specific diversity ranged between 7.2 and 17.2% in the current study and is comparable to other mosquito barcoding studies conducted in Pakistan (2.3–17.8%) [5], China (2.3–21.8%) [7], India (5.87–25.65%) [9], and Japan (4.1–12.5%) [54]. Although the effect is not known, the prominent amino acid mutation I95V, which was present is six species, has been reported from *Anopheles gambiae* as well [13].

According to morphology-based taxonomy, anophelines can be categorized into five groups: Pyretophorus (*An. subpictus*, *An. vagus*), Myzorhynchus (*An. barbirostris*, *An. nigerrimus*, *An. peditaeniatus*), Myzomyia (*An. aconitus, An. culicifacies*, *An. varuna)*, Neocellia (*An. annularis*, *An. jamesii*, *An. karwari*, *An. maculatus*, *An. pallidus*, *An. pseudojamesi*), and Neomyzomyia (*An. tessellatus*) [30]. The NJ tree for ITS2 agrees with this grouping system as it forms four separate, strongly supported clades (96–100% bootstrap value), each representing a morphology-based group. Although the *COI* phylogenetic tree is different from the ITS2 tree and does not totally agree with the morphology-based grouping system, true phylogenetic relationships need further investigation.

Only the undamaged specimens were used for morphological identifications and these were subsequently subjected to DNA barcoding. It was evident that both systems can be successfully used in species identification although subspecies-level identification could only be achieved with barcoding. Therefore, molecular characterization is a good method for both species and subspecies-level identification even with damaged specimens.

This is the first record of using both traditional and DNA barcoding to characterize several *Anopheles* species in Sri Lanka and it demonstrates the usefulness of DNA barcoding in confirming the identity of sibling species and closely related species. Further, as ITS2 confirmed *COI* findings, these two markers could be successfully used in characterizing Sri Lankan anophelines.

## Conclusions

Genetic markers *COI* and ITS2 can be successfully used in characterizing anophelines of Sri Lanka, especially in recognizing the members of the *An. subpictus* species complex. Molecular characterization data can be reliably used in situations where morphological identification fails due to damaged morphological characters. The *COI* and ITS2 sequences generated could be used as reference sequences in future mosquito identification studies. Since DNA barcoding needs advanced laboratory facilities, the use of this method in routine malaria control programmes may be restricted unless the morphological characters are uncertain or identification to subspecies level is required. Presence of the major and potential vectors of malaria in different geographical locations perhaps indicates the potential for re-emergence of malaria in the country where malaria has recently been eliminated.

## Additional files

**Additional file 1.** Anopheline species present in each study site with habitat type descriptions.

**Additional file 2.** GenBank accession numbers of *COI* and ITS2 sequences obtained during the current study and the publicly available sequence that showed highest similarity (>96% similarity) to these sequences. The fragment length of the ITS2 sequences of each species generated during the present study is also given.

## Abbreviations

*COI*: *cytochrome c oxidase subunit I*
ITS2: gene and internal transcribed spacer 2 region
BOLD: barcode of life database
K2P: Kumura-2 parameter
NJ: neighbour joining
