# Supplementary material for: Molecular characterization of Anopheline (Diptera: Culicidae) mosquitoes from eight geographical locations of Sri Lanka
Source: Malar J. 2017 Jun 2;16:234. doi: 10.1186/s12936-017-1876-y (PMC5457728; doi:10.1186/s12936-017-1876-y)
Supplement: Supplementary file 1 — Additional file 1. Anopheline species present in each study site with habitat type descriptions. [file 12936_2017_1876_MOESM1_ESM.docx]

**Additional file 1 Anopheline species present in each study site with habitat type descriptions**

| **Study site** | **Habitat type** | **Species present** |
| --- | --- | --- |
| Adikarigama | Paddy fields and dense vegetation | *An. culicifacies, An. maculatus, An. vagus* |
| Batticaloa | Coastal area with little vegetation, few paddy fields, a large fresh water storage tank and few human settlements | *An. karwari, An. pallidus, An. peditaeniatus, An. subpictus* A, *An.vagus* |
| Haldummulla | Dense vegetation, with a close by river | *An. aconitus, An. annularis An. barbirostris, An. culicifacies, An. jamesii, An. pallidus, An. peditaeniatus, An. pseudojamesi, An. subpictus* A, *An. vagus* |
| Kalmunai | Coastal area with little vegetation, few paddy fields and few human settlements | *An. pallidus, An. peditaeniatus, An. subpictus* A, *An. vagus* |
| Kattai-Adampan | Paddy fields with few human settlements | *An. barbirostris, An. jamesii, An. nigerrimus, An. peditaeniatus, An. subpictus* A |
| Matale | Dense vegetation and paddy fields | *An. jamesii, An. nigerrimus An. peditaeniatus, An.vagus* |
| Tirunelveli | Coastal area with little vegetation, paddy fields, large fresh water storage tanks and few human settlements | *An. annularis, An. culicifacies, An. jamesii, An. nigerrimus, An. pallidus, An. peditaeniatus, An. subpictus* B, *An. vagus, An. varuna* |
| Wariyapola | Little vegetation with a close by river and few human settlements | *An. barbirostris, An. jamesii, An. karwari, An. pallidus, An. peditaeniatus, An. subpictus* A, *An. tessellatus, An. vagus, An. varuna* |
