# Supplementary material for: Molecular characterization of Anopheline (Diptera: Culicidae) mosquitoes from eight geographical locations of Sri Lanka
Source: Malar J. 2017 Jun 2;16:234. doi: 10.1186/s12936-017-1876-y (PMC5457728; doi:10.1186/s12936-017-1876-y)
Supplement: Supplementary file 2 — Additional file 2. GenBank accession numbers of COI and ITS2 sequences obtained during the current study and the publicly available sequence that showed highest similarity (>96% similarity) to these sequences. The fragment length of the ITS2 sequences of each species generated during the present study is also given. [file 12936_2017_1876_MOESM2_ESM.docx]

**Additional file 2** GenBank accession numbers of *COI* and ITS2 sequences obtained during the current study and the publicly available sequence that showed highest similarity (>96% similarity) to these sequences. The fragment length of the ITS2 sequences of each species generated during the present study is also given.

| Species name | *COI* | | ITS2 | | |
| --- | --- | --- | --- | --- | --- |
|  | GenBank accessions numbers | The closest available public sequence | GenBank accessions numbers | Fragment size | The closest available public sequence |
| *An. aconitus* | KX599412-413 | HQ877378 Viet-Nam  DQ000253 Thailand | KY000680 | 461 | AY547362 Thailand  AJ626946 Sri Lanka |
| *An. annularis* | KX599414-416 | AY917197 and JN8332671 India | KY000681 | 418 | FJ526607 Sri Lanka  DQ279445 India |
| *An. barbirostris* | KX599417 | AY729982 India | *-* | - | - |
| *An. culicifacies* | KX599418-421 | KP197036 and KP197031 Sri Lanka | KY000682 | 451 | EU882735-736 India |
| *An. jamesii* | KX618709- 715 | KJ461792 Sri Lanka  JN596971 India | KY000683 | 456 | FJ 526628 Sri Lanka |
| *An. karwari* | KY196413 | KF564710 Singapore | KY196414 | 468 | FJ526635 Sri Lanka |
| *An. maculatus* | KX618716-718 | JN596972 and EU256336 India | KY000684 | 398 | KM663756, FJ526579 Sri Lanka |
| *An. nigerrimus* | KX618719- 723 | AB778799 Colombia  AB778791 Thailand | KY000685 | 506 | AB778774, AB778779 Thailand |
| *An. pallidus* | KX618724-727 | AY729974 India | KY000686 | 426 | FJ526606, JQ268282 Sri Lanka |
| *An. peditaeniatus* | KX644156-165 | JN596970 and GQ259186 India | KY000687 | 459 | AB731654 Japan |
| *An. pseudojamesi* | KX618728 | None | KY000688 | 499 | FJ526632-34 Thailand |
| *An. subpictus* species A | KX644166-181 | HQ609132 Sri Lanka  DQ 310146 India | KY000689 | 444 | KJ019331, KJ437451-452 Sri Lanka |
| *An. subpictus* species B | KX644182-183 | KJ461788-789 Sri Lanka | *-* | - | - |
| *An. tessellatus* | KX668149- 151 | AB738146 Japan | *-* | - | - |
| *An.vagus* | KX668152-163 | GQ284810 Mymmar  GQ284767 Thailand | KY000690 | 505 | KJ716079 India  AB731658 Viet-Nam |
| *An. varuna* | KX668164-168 | HQ877380 Viet-Nam  DQ149241 India | *-* | - | - |
